# A Pilot Outreach HIV Testing Project Among Homeless Adults

**DOI:** 10.3389/fpubh.2021.703659

**Published:** 2021-07-28

**Authors:** Latrice C. Pichon, Kristen Rae Rossi, Theresa Chapple-McGruder, Lisa Jane Krull, Jennifer Kmet, April L. Carswell

**Affiliations:** ^1^School of Public Health, The University of Memphis, Memphis, TN, United States; ^2^Battelle Formerly Shelby County Health Department, Memphis, TN, United States; ^3^Essence of Public Health, Washington, DC, United States; ^4^Shelby County Health Department, Bureau of Epidemiology and Infectious Diseases, Memphis, TN, United States

**Keywords:** HIV testing, homelessness, Ryan White part A, behavioral model for vulnerable populations, service utilization

## Abstract

**Background:** The Memphis metropolitan statistical area (MSA) represents a Deep Southern U.S. city disproportionally affected by the ongoing transmission of new HIV cases as well as those diagnosed in late-stage disease. This region is a subset of nine states, including Memphis, Tennessee (project site), driving the epidemic in the United States. Memphis ranks 4th among all U.S. MSAs for new HIV infections and has been identified in the CDC's Ending the HIV Epidemic Initiative as a high HIV burden geographic focus area. The Memphis Ryan White Part A Program conducted a pilot project among adults seeking services in Memphis emergency and transitional housing shelters to offer on-site, rapid HIV testing. In this paper we describe the results from this aforementioned pilot study, including the rate of HIV test acceptance and potential factors associated with a history of HIV testing in Memphis.

**Methods:** Community-engaged research approaches were employed *via* a partnership between the local health department, a federally qualified faith-based health center, and an academic university. An interviewer-administered survey to measure potential factors associated with HIV testing history and voluntary HIV testing services were offered to adults living in transitional housing establishments. Bivariate chi-square analyses were performed to determine the association between predisposing, enabling, and need variables with HIV testing history in the past 12 months.

**Results:** Survey respondents (*n* = 109) were mostly cisgender male (*n* = 96; 88.1%), African American (*n* = 79; 72.5%) and reported engaging in condomless sex in the past 12 months (*n* = 55; 50.5%). Acceptability and uptake of HIV testing was high (*n* = 97; 89.0%).

**Conclusions:** Implementing rapid HIV testing programs outside of traditional health care settings is a strategy that can be used to engage high-risk individuals and those unaware of their HIV status to get tested. To our knowledge, this study represents the first that documents HIV testing acceptance rates offered outside of traditional health care settings for homeless and transitionally housed adults in a Deep Southern state.

## Background

According to the Centers for Disease Control and Prevention (CDC), an estimated one-in-seven persons living with HIV in the United States in 2018 are unaware they are infected (1). The National HIV/AIDS Strategy (NHAS) cites this estimate as a challenge to reducing HIV transmission and acquisition since persons unaware of their status may unintentionally expose others (2). In 2010, the NHAS first recommended early testing and treatment as a primary strategy for reducing HIV incidence, and the updated NHAS 2020 Strategy includes specific guidelines, suggesting all HIV-negative people at high risk for infection should be tested at least annually (3). The NHAS 2020 also calls for ongoing support for the Ryan White HIV/AIDS Program (RWHAP), which works with cities, states, and local community-based organizations to provide a comprehensive system of care for those who do not have sufficient health care coverage or financial resources to cope with HIV disease (2). While the majority of RWHAP funds support primary medical care and essential support services, activities related to “early intervention services” are allowed to facilitate access to the HIV care system using HIV testing, referral services, health literacy, and care linkage to bridge medication access and treatment adherence (4).

Southern states accounted for ~51% of all people in the US with HIV at the end of 2018 (5). The Memphis Metropolitan Statistical Area (MSA) represents a Southern city disproportionally affected by the ongoing transmission of new HIV cases as well as those diagnosed in late-stage disease. In 2018, the Memphis MSA represented the third-highest rate of new HIV diagnoses in the United States (6). In 2007, The Memphis Area RWHAP was first awarded Part A and Minority Initiative AIDS funding by the Health Resources and Services Administration (HRSA) to provide primary medical care and essential support services for people living with HIV who were uninsured or underinsured within a seven-county area surrounding Memphis, spanning across Tennessee, Mississippi, and Arkansas (7). By 2011, the program had grown to serve over 4,500 persons living with HIV or AIDS; however, an estimated additional 2,000 individuals were infected but unaware of their positive status at the end of 2010 (8). In response to the large estimates of those unaware of their HIV status as well as HRSA requirements mandating early intervention strategies for all RWHAP nationwide, the Memphis Area RWHAP identified several vulnerable populations on which to focus early detection and treatment resources. These populations included homeless and transitionally housed populations (9). This strategy was corroborated by results from an annual point-in-time survey conducted by the Memphis and Shelby County Department of Housing and Urban Development, where 2% of homeless adults self-identified an HIV positive status (10). Furthermore, no HIV testing services were provided specifically for the homeless in concentrated, routine outreach efforts in Memphis.

Homeless and unstably housed individuals represent subpopulations at high risk for HIV infection, due in part to higher prevalence of alcohol dependence, illegal substance use, and sexual risk behaviors (11–13). Research estimates of HIV prevalence among unstably housed populations vary widely by geographic region and sampling methodologies. Further, a meta-analysis that included 43 studies conducted in the U.S. found prevalence rates of HIV among those with unstable housing ranged from 0.3 to 21% (14). Studies throughout the U.S. have concluded that community-based rapid HIV testing is feasible, acceptable, and effective for homeless populations, but these studies have not been documented in Southern states, and few describe the rates of HIV test acceptance through on-site screening programs (15, 16). The Memphis RWHAP conducted a pilot project among adults seeking services in Memphis emergency and transitional housing shelters to offer on-site, rapid HIV testing as well as an interviewer-administered survey to measure potential factors associated with HIV testing history. In this paper we describe the results from this aforementioned pilot study, including the rate of HIV test acceptance and potential factors associated with a history of HIV testing.

## Methods

A total of 116 adults receiving services from emergency and transitional housing providers in Memphis were recruited using posted flyers in facilities and constituted a cross-sectional, convenience sample. Inclusion criteria required participants to report they were currently sleeping at a housing shelter or had no place to sleep and were at least 18 years of age. Upon completion of a written informed consent form, participants were asked to complete an interviewer-administered survey and then were offered voluntary HIV testing services. Survey data collected from seven participants were excluded due to incomplete data or non-eligibility, resulting in a final sample of 109 participants for data analysis. Participants received a $5 gift card to a local grocery store for completing the survey.

Application of the Behavioral Model for Vulnerable Populations with relation to use of HIV testing services guided the implementation of the pilot study (17, 18). This model addresses predisposing, enabling, and need factors relevant to understanding the health and health-seeking behaviors of vulnerable populations and has been applied in other studies to identify challenges in obtaining needed services for the homeless (19, 20). Use of HIV testing services was documented in two ways: the interviewer-administered survey included a question to document self-report of HIV testing within the past 12 months, and interviewers also recorded whether or not the participant accepted or refused voluntary HIV testing services following completion of the survey.

Assessment of predisposing variables included socio-demographic factors (gender, age, race, and education), duration of homelessness, history of incarceration, alcohol abuse or dependence, and general physical and mental health status. We also used the Alcohol Use Disorders Identification Test—Consumption (AUDIT-C), a validated (i.e., AUROC.891), three-item screening tool, to assess alcohol abuse or dependence (21). To evaluate general physical and mental health status, a standardized measure included self-report response options of excellent, very good, fair, or poor. Enabling factors were defined as those that affect health education opportunities and HIV-risk assessment. These variables included current health insurance coverage, when and where the participant last accessed a doctor, and whether or not the participant had one person considered as a personal doctor or health care provider. To evaluate factors related to need for HIV testing, participants were asked to describe self-perceived susceptibility and risk behaviors. Perceived susceptibility for HIV infection was evaluated using a scale from zero to four, where zero represented the participant was “not worried at all” about being infected, and four indicated the participant was “extremely worried.” This scale has previously been used in research studies with homeless adults and has been assessed for internal reliability and predictive validity (22). The Fogg HIV Screening Questionnaire was employed to assess HIV-risk behaviors in the 12 months prior to the survey. This instrument has been evaluated for reliability (Cronbach's 0.72–0.90; test-retest 0.56–0.86) within homeless populations; it includes eight self-report questions to assess specific sexual risk behaviors and two additional questions to assess injection drug use and diagnosis of a sexually transmitted infection (23).

The survey instrument was qualitatively pilot tested with a subset of the priority population. Participants were asked five brief questions about the difficulty, similarity, length of the survey, comfort level answering the questions, and suggestions to improve the survey. Data entry was performed by the lead epidemiologist and verified by the graduate assistant. Use of HIV testing services are described as a percent of participants (a) ever tested for HIV, (b) tested within the past 12 months, and (c) accepting HIV testing at the time of the survey. Bivariate chi-square analyses were performed to determine the association between predisposing, enabling, and need variables with the individual's HIV testing history in the past 12 months. All analyses were performed using the SAS Statistical Analysis package (SAS Institute version 9.3).

A community engaged partnership approach was employed between a local health department, university, and a community federally qualified health center to develop the study design, co-create the survey instrument, administer surveys, and disseminate study findings (24). The community health center was identified as a viable research partner given the range of medical and supportive services it provided for persons living with HIV/AIDS, prior experience in providing outreach HIV testing services for the homeless population, and long-standing partnership with the local health department. Each member of the data collection team received training in research ethics and study procedures, participated in interview role plays, and observed in the field to maintain quality control and receive immediate corrective feedback. Approval of an Institutional Review Board was obtained from the University of Memphis (Protocol #2049). Free confidential HIV testing was offered to all participants but not required for survey participation.

## Results

Among the 109 participants, the majority of our study's respondents were male (*n* = 96; 88.1%), African American or Black (*n* = 79; 72.5%), had a history of incarceration (*n* = 91; 83.5%), and reported their overall physical health (*n* = 77; 70.6%) and mental health (*n* = 79; 72.5%) as either good, very good, or excellent. Participants between 40–49 years (*n* = 41; 37.6%) and 50+ years (*n* = 39; 35.8%) accounted for the two largest age groupings. Almost half of the participants reported short-term homelessness, where 47.7% (*n* = 52) indicated they had been homeless for <12 months. Less than half screened positive for alcohol dependence or abuse (*n* = 48; 44.0%) on the AUDIT-C tool. Sex without a condom (*n* = 55; 50.5%), sex while drunk or high on drugs (*n* = 52; 47.7%), and sex with an unknown person (*n* = 26; 23.9%) emerged as the three most prevalent risk behaviors in the 12 months preceding the survey administration. Chi-square analyses found no significant associations between predisposing, enabling, and need variables with HIV testing history in the past 12 months (Table 1).

**Table 1:** HIV testing history by predisposing, enabling and need variables.

	**No HIV test in past 12 months (** ***n*** **=** **77)**		**Tested for HIV in past 12 months (** ***n*** **=** **32)**		**Total Sample (** ***n*** **=** **109)**		
Variable^*^	***N***	**%**	***N***	**%**	***n***	**χ^**2**^-statistic**	***P*-value**
**Predisposing variables**							
Gender						0.59	*0.44*
Male	69	71.9%	27	28.1%	96		
Female	8	61.5%	5	38.5%	13		
Age						3.85	*0.15*
19–39	19	65.5%	10	34.5%	29		
40–49	26	63.4%	15	36.6%	41		
50+	32	82.1%	7	17.9%	39		
Race						2.92	*0.09*
Black/African American	52	65.8%	27	34.2%	79		
White	24	82.8%	5	17.2%	29		
Duration of homelessness						0.05	*0.83*
<12 months	36	69.2%	16	30.8%	52		
1 + years	37	71.2%	15	28.8%	52		
Education						2.60	*0.27*
Less than GED/high school diploma	21	75.0%	7	25.0%	28		
High school diploma or equivalent	33	63.5%	19	36.5%	52		
Some college/graduate	23	79.3%	6	20.7%	29		
History of Incarceration						1.67	*0.20*
Yes	62	68.1%	29	31.9%	91		
No	15	83.3%	3	16.7%	18		
Alcohol dependence						0.02	*0.89*
Yes	34	70.8%	14	29.2%	48		
No	39	69.6%	17	30.4%	56		
Physical health						2.20	*0.14*
Fair, Poor	25	80.6%	6	19.4%	31		
Good, Very Good, Excellent	51	66.2%	26	33.8%	77		
Mental health						1.07	*0.30*
Fair, Poor	19	63.3%	11	36.7%	30		
Good, Very Good, Excellent	58	73.4%	21	26.6%	79		
**Enabling variables**							
Health insurance coverage						0.14	*0.71*
Yes	19	67.9%	9	32.1%	28		
No/Unknown	58	71.6%	23	28.4%	81		
Last doctor visit						2.63	*0.11*
<12 months ago	52	65.8%	27	34.2%	79		
1+ years ago	23	82.1%	5	17.9%	28		
Regular source of care						0.08	*0.77*
Yes	29	69.0%	13	31.0%	42		
No	48	71.6%	19	28.4%	67		
**Need variables**							
Perception of HIV risk						0.06	*0.97*
Not at all	36	70.6%	15	29.4%	51		
Little/slightly/somewhat	17	70.8%	7	29.2%	24		
Extremely	19	73.1%	7	26.9%	26		
HIV-risk behaviors (in the past 12 months)^**^						1.85	*0.60*
Sex without a condom	40	72.7%	15	27.3%	55		
Sex while drunk or high on drugs	35	67.3%	17	32.7%	52		
Sex with an unknown person	19	73.1%	7	26.9%	26		
Five or more sex partners	9	56.3%	7	43.8%	16		
Used a needle to inject drugs	6	75.0%	2	25.0%	8		
Sex with IDU (IV Drug User)	5	71.4%	2	28.6%	7		
Diagnosis of STD	2	50.0%	2	50.0%	4		
Traded sex for drugs or money or something else needed	2	66.7%	1	33.3%	3		
Sex with a man who has had sex with a man (MSM)	2	100.0%	0	0.0%	2		
Sex with someone who is HIV positive	1	100.0%	0	0.0%	1		
**Total**	**77**	70.6%	**32**	29.4%	**109**		

* *Missing and unknown data are excluded from chi-square statistic tests; thus, variable category totals may not equal column totals*.

** *HIV-risk behaviors are not exclusive and participants had the option to elect more than one response. Chi-square statistic test only included the four highest frequency risk behavior categories, including sex without a condom, sex while drunk or high on drugs, sex with an unknown person, and five or more sex partners. Bold values are the total number of participants i.e., 77 reporting no HIV test in past 12 months, 32 reporting HIV test in past 12 months, and 109 total sample. Italic values in the last column are the non-significant p-values*.

The large majority of participants accepted HIV testing following survey administration (*n* = 97; 89.0%). While 73.4% (*n* = 80) participants reported ever having an HIV test in the past, only 29.4% (*n* = 32) had been tested for HIV in the 12 months prior to the survey. Nearly three-quarters of the study participants reported no health insurance coverage, or they didn't know if they had coverage; however, 72.5% (*n* = 79) reported seeing a doctor in the past 12 months. Among the 79 participants self-reporting a doctor visit in the 12 months prior, only 34.1% (*n* = 27) reported they had been tested for HIV during the same time period. For those reporting a doctor's visit or HIV test within the past 12 months, the type of facility is displayed in Table 2. A hospital or emergency room accounted for both the largest number of participants reporting the location of last doctors' visit (*n* = 25; 31.6%) and last HIV test (*n* = 7; 21.9%). The second and third most frequently cited locations for last doctors' visit included public health clinics/health departments (*n* = 15; 19.0%) and the Veterans Affairs hospital (*n* = 14; 17.7%). Public health clinics/health departments accounted for the second most frequently cited location among participants reporting an HIV test in the past 12 months (*n* = 7; 21.9%), while outreach mobile clinics accounted for the third most frequently cited location (*n* = 5; 15.6%).

**Table 2:** Site of last doctor visit and Last HIV test within 12 months prior to survey^*^.

	**Participants reporting doctor visit in past 12 months (** ***n*** **=** **79)**		**Participants reporting HIV test in past 12 months (** ***n*** **=** **32)**	
**Site**	***N***	**%**	***N***	**%**
Hospital/emergency room	25	31.6%	7	21.9%
Public health clinic/health department	15	19.0%	7	21.9%
Veterans affairs	14	17.7%	0	0.0%
Private doctor office	10	12.7%	1	3.1%
Outreach mobile clinic	6	7.6%	5	15.6%
Prison/jail	2	2.5%	3	9.4%
Shelter	2	2.5%	0	0.0%
Drug rehab facility	0	0.0%	1	3.1%
Blood bank	0	0.0%	2	6.3%
Unknown/other site	5	6.3%	6	18.8%

* *P-value > 0.05*.

## Discussion

Implementing rapid HIV testing programs outside of traditional health care settings is a strategy to target high-risk individuals and those unaware of their HIV status. To our knowledge, this study represents the first that documents HIV testing acceptance rates offered outside of traditional health care settings for homeless and transitionally housed adults in a Southern state. Two studies have demonstrated the rate of acceptance for rapid HIV testing in community-based outreach programs and ranged from 60% to 75%, varying by geography and venue for recruitment (15, 25). The acceptance of HIV testing following our survey administration was higher than expected based upon the results of the aforementioned studies. When offered the option to receive an HIV test following completion of our survey interview, almost 90% agreed to be tested. This acceptance rate would seem to indicate that rapid HIV testing might be well-accepted among this population given the option onsite at locations offering services to homeless and transitionally housed adults.

Almost three-quarters of our participants had a doctor visit within the 12 months prior to our survey, but only 34% of these individuals had also been tested for HIV during this same period. This disparity, coupled with the high acceptance of HIV testing in this pilot outreach testing project, potentially demonstrates missed opportunities to implement early testing strategies supported by the NHAS in a variety of health care settings. The majority of participants reported the site of last doctor visit was received at hospitals/emergency rooms or public health clinics, indicating these venues as important points of health care access for this population. For our pilot project we recruited participants from venues serving homeless populations, such as emergency shelters, transitional housing, and drop-in centers where meals were provided. The high acceptance rates of HIV testing following the survey administration indicates these sites as potential venues to provide mass HIV testing outside of traditional health care access points.

Although no significant associations were identified between or among any predisposing, enabling, or need variables and a history of HIV testing in the 12 months prior to the survey, the reported high-risk behaviors are not without warrant. Over two-thirds of the sample reported engaging in at least one high-risk behavior in that 12-month time period. The most frequently cited behaviors within the past 12 months included engaging in sexual activity with unknown persons, being under the influence of alcohol or drugs, and having sex without using condom. High risk behaviors such as the aforementioned may be attributed to survival sex to secure goods, services, and maintain housing (26). Nevertheless, these results still highlight the need to prioritize routine and early testing strategies for this at-risk population.

This study is not without limitations. With respect to assessing a history of HIV testing, we did not document if other health care providers offered testing for regular office visits and if our participants declined. Likewise, homeless adults from these facilities self-selected to participate in the survey and thus may view issues related to HIV testing differently from non-participants. We did not conduct *post hoc* analysis to assess the psychometric properties of the survey. However, we administered the survey to a sub-sample from the priority population to qualitatively assess readability and comfortability. Finally, we did not assess same sex behaviors and survival sex among our mostly cisgender male sample, which could potentially provide additional risk for HIV acquisition.

## Conclusions

Offering on-site, rapid HIV testing for unsheltered populations is paramount as the Memphis community works toward developing a local plan for the U.S. Ending the HIV Epidemic Initiative (27). Housing and economic instability will remain barriers to reaching benchmarks for prevention and testing pillars. These two social determinants of HIV are associated with viral suppression (28). Permanent housing for those living with HIV increases medication adherence, retention in care, and access to supportive services to better achieve viral suppression (29). Similarly, housing stability could facilitate other prevention goals such as testing and access and adherence to PrEP for those engaging in condomless sex. The current pilot project demonstrates acceptability of non-traditional testing and potential factors to consider as facilitators of HIV testing behaviors in emergency shelters.

With the recent onset of COVID-19 and the subsequent effect that the pandemic will have on housing and economic instability, it remains essential to maintain HIV testing outside of traditional health care settings (30). More and more Black Americans living in the South will no longer have reliable and consistent employment to maintain stable housing or health insurance coverage to seek HIV preventive services. Lack of Medicaid expansion to an already vulnerable southern city alongside threats of disruption of coverage under the Affordable Care Act will exacerbate existing disparities (31, 32). Furthermore, our health care systems will likely see an uptick in use of hospital or emergency rooms for primary health care for mostly preventable illness post COVID-19 (33, 34).

The findings from this pilot HIV testing intervention in non-traditional settings has significant public health implications for future prevention and testing goals to curb the epidemic in the South. The high acceptability and uptake of HIV screening support the utility of community-engaged approaches and strategies to successfully partner with the local public health department and federally qualified health centers to increase HIV testing rates among particularly at-risk populations. It also provides context to the feasibility of offering testing in transitional housing locations with limited disruption to facility operations. Finally, the practicality of involving frontline staff in the research design and implementation of an HIV testing research study strengthens the acceptability of community-based projects and community participation. We recommend future studies use rigorous study designs with a comparison group, follow participants overtime, and conduct exploratory analyses to understand the impact of testing adherence and housing status.

## Data Availability Statement

The dataset generated and analyzed during the current study are not publicly available due to our lack of non-data sharing agreement during the acquisition of data but are available from the corresponding author on reasonable request.

## Ethics Statement

The studies involving human participants were reviewed and approved by The University of Memphis Institutional Review Board. The participants provided their written informed consent to participate in this study.

## Author Contributions

LP, KR, TC-M, LK, JK, and AC made substantial contributions to the conception of the work, co-wrote sections of the paper and have read and approved the final manuscript. All authors have agreed both to be personally accountable for the author's own contributions and to ensure that questions related to the accuracy or integrity of any part of the work are appropriately investigated.

## Conflict of Interest

The authors declare that the research was conducted in the absence of any commercial or financial relationships that could be construed as a potential conflict of interest.

## Publisher's Note

All claims expressed in this article are solely those of the authors and do not necessarily represent those of their affiliated organizations, or those of the publisher, the editors and the reviewers. Any product that may be evaluated in this article, or claim that may be made by its manufacturer, is not guaranteed or endorsed by the publisher.

## Abbreviations

MSA: metropolitan statistical area
CDC: Centers for Disease Control and Prevention
NHAS: National HIV/AIDS Strategy
RWHAP: Ryan White HIV/AIDS Program
HRSA: Health Resources and Services Administration
AUDIT-C: alcohol use disorders identification test.
